# Therapeutic efficacy of artemether-lumefantrine on treatment of uncomplicated *Plasmodium falciparum* mono-infection in an area of high malaria transmission in Zambia

**DOI:** 10.1186/1475-2875-13-430

**Published:** 2014-11-17

**Authors:** Busiku Hamainza, Freddie Masaninga, Hawela Moonga, Mulenga Mwenda, Pascalina Chanda-kapata, Victor Chalwe, Emmanuel Chanda, Mulakwa Kamuliwo, Olusegun Ayorinde Babaniyi

**Affiliations:** Ministry of Health, National Malaria Control Centre, Chainama Hospital College Grounds, off Great East road, P.O. Box 32509, Lusaka, Zambia; Liverpool School of Tropical Medicine, Vector Group, Pembroke Place, Liverpool, L3 5QA UK; Akros Global Health, Lusaka, Zambia; World Health Organization, Lusaka, Zambia; Ministry of Health, Head-quarters, Ndeke House, Lusaka, Zambia; Maina Soko Military Hospital, Zambia Army Head-quarters, PO Box 31931, Lusaka, Zambia

**Keywords:** Antimalarial treatment, Artemether lumefantrine, Efficacy, Safety, All ages, Zambia

## Abstract

**Background:**

Anti-malarial drug resistance continues to be a leading threat to ongoing malaria control efforts and calls for continued monitoring of the efficacy of these drugs in order to inform national anti-malarial drug policy decision-making. This study assessed the therapeutic efficacy and safety of artemether-lumefantrine (AL)(Coartem®) for the treatment of uncomplicated *Plasmodium falciparum* malaria in two sentinel high malaria transmission districts in the Eastern Province of Zambia in persons aged six months and above, excluding women aged 12 to 18 years.

**Methods:**

This was an observational cohort of 176 symptomatic patients diagnosed with uncomplicated *Plasmodium falciparum* mono-infection. A World Health Organization (WHO)-standardized 28-day assessment protocol was used to assess clinical and parasitological responses to directly observed AL treatment of uncomplicated malaria. DNA polymerase chain reaction (PCR) analysis for molecular markers of AL resistance was conducted on positive blood samples and differentiated recrudescence from re-infections of the malaria parasites.

**Results:**

All patients (CI 97.6-100) had adequate clinical and parasitological responses to treatment with AL. At the time of enrolment, mean slide positivity among study participants was 71.8% and 55.2% in Katete and Chipata, respectively. From a mean parasite density of 55,087, 98% of the study participants presented with zero parasitaemia by day 3 of the study. Fever clearance occurred within 24 hours of treatment with AL. However mean parasite density declines were most dramatic in participants in the older age. No adverse reactions to AL treatment were observed during the study.

**Conclusion:**

AL remains a safe and efficacious drug for the treatment of uncomplicated *Plasmodium falciparum* malaria in Zambia, endemic for malaria, with some provinces experiencing high transmission intensity. However, the delayed parasite clearance in younger patients calls for further sentinel and periodical monitoring of AL efficacy in different areas of the country.

## Background

Malaria is a parasitic infection endemic in Zambia. It is reported to be among the 10 top causes of morbidity and mortality in health facilities in the country [1]. A 50% decline in malaria cases and deaths was observed between the years 2000 to 2010 [2] and this was attributed to improved funding, technical assistance and scale up of cost-effective preventive and curative interventions [3]. However, continued success in controlling malaria continues to be threatened by the development of resistance to anti-malarial medicines, as evidenced in Zambia with resistance development to chloroquine and sulphadoxine-pyrimethamine [4, 5]. In this regard, following recommendations by the World Health Organization (WHO) [6, 7], Zambia reviewed its malaria treatment policy in 2003 removing Chloroquine (CQ) as the first-line treatment for uncomplicated malaria and replaced it with artemisinin combination therapy (ACT), with the drug of choice being the co-formulated artemether-lumefantrine (AL) (20 mg artemether and 120 mg lumefantrine) [5]. AL is recommended for the treatment of uncomplicated malaria because of its rapid reduction of parasite load as a result of the action of the artemisinin component and continued elimination of residual parasites by lumefantrine which results in a rapid clinical response [6, 7]. ACT may reduce the development of parasite resistance subsequently by contributing to the reduction of malaria transmission [8].

The policy change from CQ to AL in 2003 was well accepted by users on account of its proven therapeutic efficacy even against multi-drug resistant parasites, optimal tolerability and safety profile. However, continued use of these anti-malarial medicines including, AL is threatened by the development of parasite resistance. However, currently, there are no alternative anti-malarial drugs available with proven therapeutic efficacy even against multi-drug resistant parasites, optimal tolerability and safety profile as artemisinin-based combinations [9]. In order to ensure effective malaria case management, it may be imperative to preserve the user-life of ACT. For this reason, National Malaria Control Programmes should conduct regular therapeutic efficacy testing of anti-malarial drugs to provide timely, relevant and reliable information to guide malaria treatment policy development.

This study was conducted to provide efficacy and safety data on artemether-lumefantrine following a standard WHO 28-day follow-up therapeutic efficacy and safety protocol to guide the Zambian anti-malarial treatment policy.

## Methods

### Study design

This was a one-arm prospective study conducted in May 2012. The study assessed clinical and parasitological responses after administration of anti-malarial treatment with AL to eligible patients aged six months (>5kgs weight) and above, excluding women of the age group (12 to 18 years) suffering from uncomplicated falciparum malaria.

### Study site

**Figure 1:**
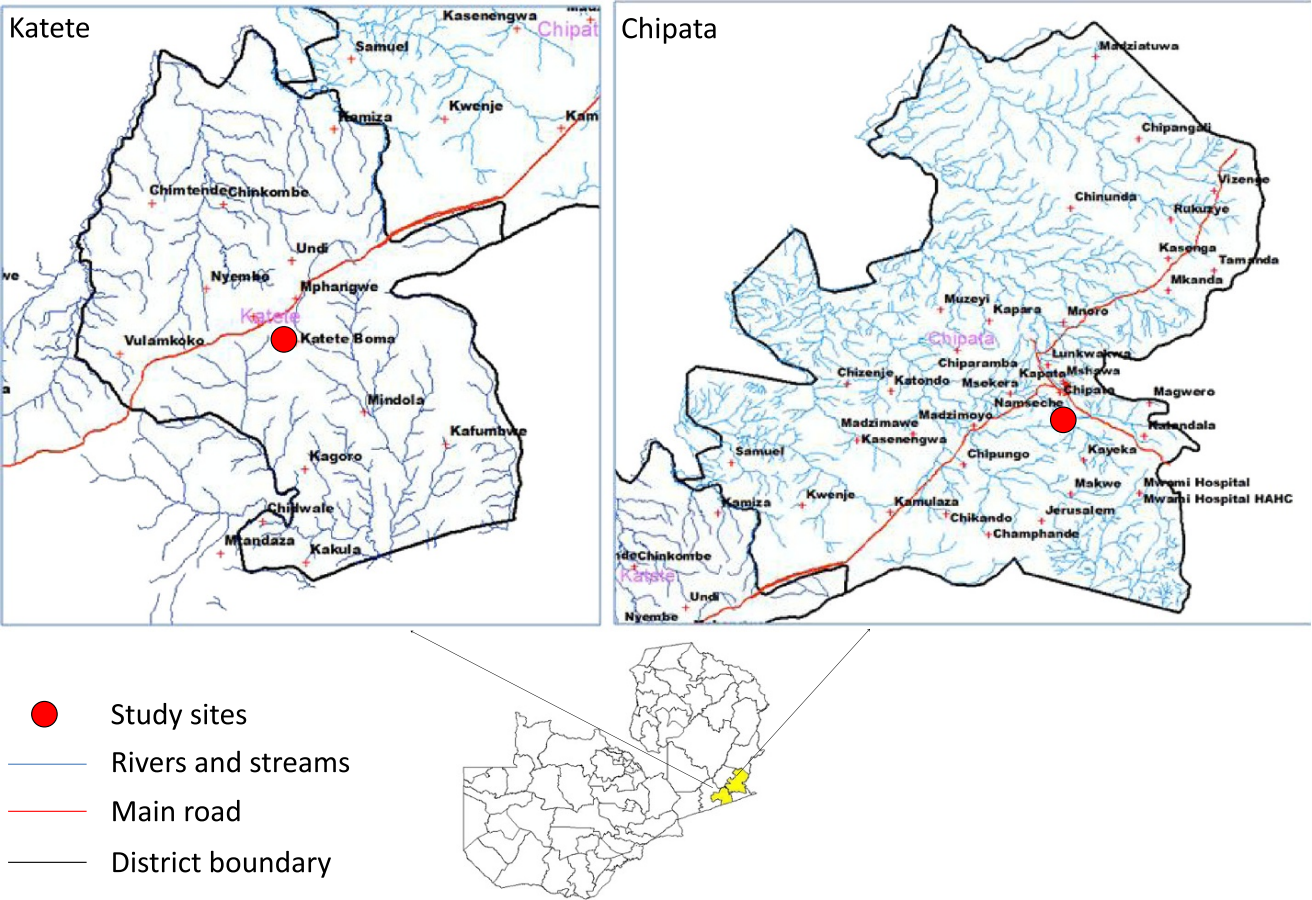
**Location of districts and study sites where the study was conducted.**

The study was conducted in two primary health facilities located in Chipata and Katete districts of Eastern Province in Zambia (Figure 1). These sites were selected based on their malaria epidemiological and geographic profile. The selected study sites had functional laboratories equipped with optimal microscopy services for malaria diagnosis and were situated near a second level district hospital for referrals of severe malaria case management if required.

### Study population

The population consisted of consenting patients with uncomplicated *Plasmodium falciparum* malaria infection seeking care at the selected study primary health care facilities who were aged six months (>5kgs weight) and above, excluding women of the age group 12 to 18 years as requesting this age group to take a pregnancy test and initiate contraception is not acceptable in the local context. All enrolled patients were treated with AL on site as directly observed treatment and monitored for 28 days, as per recommendation for evaluating clinical and parasitological response for drugs such as AL that have a half-life of less than seven days [10]. Health care services including follow-up for any illness related to malaria were provided free of charge to the study patients regardless of treatment outcome.

### Sample size

Assuming a 5% treatment failure rate to AL, a 95% confidence level and a precision around the estimate of 5%, 73 patients were targeted as a minimum for inclusion into the study. With a 20% increase to allow loss to follow-up and withdrawals during the 28-day follow-up period, 87 patients were planned to be included into the study. The study recruited a total of 177 patients overall.

### Inclusion criteria

Symptomatic patients aged six months (>5kgs weight) and above, excluding women of the age group 12 to 18 years, self-presenting to health facilities with uncomplicated malaria due to mono-infection of *P. falciparum* detected by microscopy at parasitaemia of 1,000 to 200,000/μl asexual forms, axilliary temperature ≥37.5°C, willing to comply with the study protocol for the duration of the study were included. A detailed inclusion criteria is provided elsewhere [10].

### Exclusion criteria

Patients with general danger signs or signs of severe falciparum malaria*;* unable to drink, or breast feed (in case of children), severe vomiting; reported history of convulsion seven days prior to patient contact; presence of lethargy or decreased consciousness; inability to sit or stand, were all excluded. Patients who failed to complete treatment due to persistent vomiting of the treatment or failed to attend scheduled visits during the first three days or withdrew their consent were also excluded. A detailed list of exclusion criteria is provided elsewhere [10].

### Follow-up and loss to follow up

Parents or guardians of children were instructed to return to the health centre at any time if they had any general danger signs as described under exclusion criteria above. The study team made home visits as follow ups for study participants that were late for their scheduled visits. Patients who failed to return on days 1 and 2 and missed one dose of the treatment or enrolled patients who could not attend scheduled visits were considered lost to follow up (LFU) and excluded from the final analysis.

### Anti-malarial treatment

AL was obtained from WHO and administered by a qualified Medical Officer following a treatment regime of two daily doses for three days based on the patient’s weight [7]. The day a patient was enrolled and received the first dose of AL was designated ‘Day 0 or D0’. Enrolled patients were observed for a minimum of 30 minutes after treatment to ensure that they did not vomit the drugs. Patients with persistent vomiting were excluded from the study and immediately referred to the district hospital for appropriate management. A case report form was kept for recording adverse events. Patients with fever over 38°C were treated with paracetamol or any available antipyretic. Parents or guardians were instructed in the use of tepid sponging for children under five years of age. Patients were advised not to take herbal remedies during the study to avoid effects that would confound interpretation for findings.

### Classification of responses to treatment

On the basis of parasitological and clinical outcome of treatment with AL, patients were classified according to the WHO definition of therapeutic responses [10].

### Early treatment failure (ETF)

The development of danger signs for severe malaria on days 1, 2, or 3 in the presence of parasitaemia; parasitaemia on day 2 higher than the day 0 count irrespective of axilliary temperature; parasitaemia on day 3 with axilliary temperature ≥37.5°C; parasitaemia on day 3 ≥ 25% of count on day 0.

### Late clinical failure (LCF)

The development of danger signs for severe malaria after day 3 in the presence of parasitaemia, without previously meeting any of the criteria of ETF; presence of parasitaemia and axilliary temperature ≥37.5°C or history of fever on any day from day 4 to day 28, without previously meeting any of the criteria of ETF.

### Late parasitological failure (LPF)

The presence of parasitaemia on any day from day 7 to day 28 and axilliary temperature <37.5°C, without previously meeting any of the criteria for ETF or LCF.

### Adequate Clinical and Parasitological Response (ACPR)

It is the absence of parasitaemia on day 28 irrespective of axilliary temperature without previously meeting any of the criteria for ETF, LTF, or LPF. The secondary outcomes were fever clearance rate; proportion of patients who had fever cleared on days 1, 2, and 3. Parasite clearance rate: proportion of patients with negative thick blood film smears on days 1, 2, and 3; Gametocyte carriage: proportion of patients with gametocytes during the course of the study.

### Genotyping of malaria parasites

Genotype analysis (nested PCR amplification) was conducted at the National Malaria Control Centre molecular laboratory based on genetic diversity among the malaria parasite genes *msp1, msp2* and *glurp.* The technique differentiated “recrudescence” from a newly acquired infection through a comparison of pre- and post-parasite strain genotype profiles. Details of the protocol used in this analysis are described in detail elsewhere [11].

### Data analysis

Data from both clinical and parasitological assessments from the case report for each study participant were entered into the WHO standardized Microsoft excel data collection form [10]. This form was used both for data management and analysis. Additional analysis was conducted with Microsoft excel. All data was independently double blind entered.

### Ethical considerations

Approval to conduct the study was obtained from the Tropical Diseases Research Centre ethics committee based in Ndola, Zambia. Permission to conduct the study was obtained from the Ministry Of Health. Informed consent was sought from all study participants. Guardians provided assent for the participation of the persons under the consenting age. They were asked to read or have read to them, understand and sign/thumbprint an informed consent form. The consent was available in English and explained in vernacular to the patient, parent or guardian. The benefits and potential risks were explained and a signature requested on the consent form. Confidentiality was maintained through ensuring all patients’ information used unique identifiers. All data collection forms were stored in locked files.

## Results

### Patient characteristics

A total of 142 and 230 eligible patients from Katete and Chipata, respectively, were screened during the study. The slide positivity rate was 71.8% (102/142) and 55.2% (127/230) respectively for Katete and Chipata, which translated into an overall slide positivity of 61.6% (229/372) for both study sites. Following the eligibility requirements, a total of 177 (88 Katete and 89 Chipata) were enrolled into the study (Table 1). Due to one loss to follow-up, the total analysable population was 176 (Table 1), which consisted of 49.7% female participants. In the study there were 51% (91/176) under-five participants, while 36% (63/176) comprised of patients between the age of 5 and 15. The remaining study participants were above the age of 15 (13%( 23/176)). The under-fives enrolled population had the highest mean axilliary temperature as well as parasite densities when compared to the higher age groups (Table 2).

**Table 1:** **Number of patients assessed and loss for follow up at each scheduled visit**

Day of follow up	0	1	2	3	7	14	21	28
**Patients assessed at visit**	176	173	172	172	172	171	171	171
**Loss to follow up**	0	3	1	0	0	1	0	0

**Table 2:** **Summary of patient characteristics**

Age range	Enrolled	Weight (KGs)*	History of fever*		Temperature (°C)*	Parasite density per μl*	Gametocytes*
			Yes	No			
**Under 5 yrs**	91 (51%)	11.9	89 (97.8%)	2 (0%)	38.3	67091.6	0 (0%)
**5-15 yrs**	63 (36%)	22.2	63 (100%)	0 (0%)	38.2	43987.4	0 (0%)
**Over 15 yrs**	23 (13%)	52.2	22 (95.7%)	1 (0%)	37.5	39607.6	1 (0%)
**ALL ages**	177 (100%)	20.8	174 (98.3%)	3 (1.7%)	38.1	55087.3	1 (0.5%)

*Mean estimates from Day 0 of the study.

### Primary study outcomes

**Figure 2:**
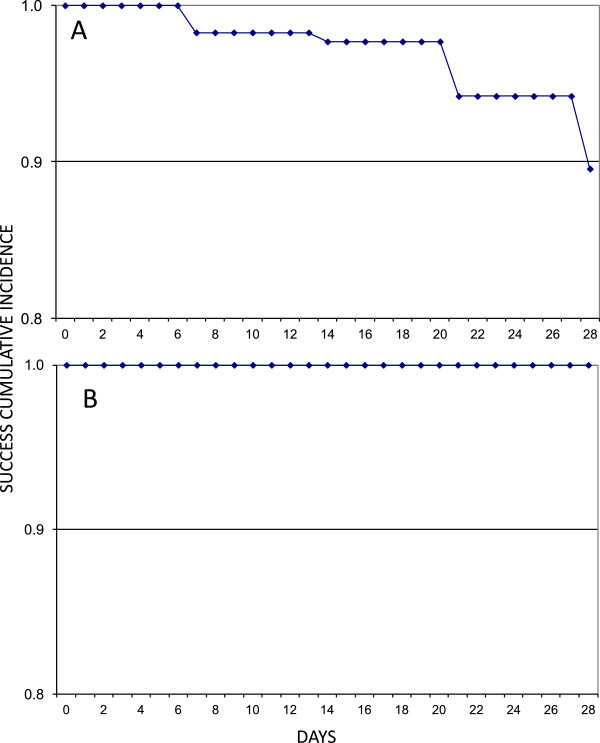
**Kaplan Meier curves showing treatment success cumulative proportion for the population under study for artemether lumefantrine up to day 28 of follow-up ((A) PCR-uncorrected and (B) PCR-corrected)).**

A total of 171 evaluable patients were assessed up to day 28. Five patients were lost to follow up during the study period. Analysis of PCR uncorrected data estimated adequate clinical and parasitological response (ACPR) as 89.5% (CI 83.9 - 93.6) in patients treated with AL. No patients showed early treatment failures (ETF), while late clinical failure (LCF) was reported in 6 study participants (3.5%(CI 1.3, 7.5)). Late parasitological failure (LPF) was observed in 12 (7.0% (CI 3.7, 11.9)) of the evaluated study population (Table 3). All reported treatment failures were observed in the age groups under the age of 15. The Katete site with 9 (i.e., seven below five years of age and two aged between five and fifteen) had more LPF than Chipata with three all aged between five and fifteen). Three LCF were reported in each of the two sites. In Katete all the participants were aged below five, whereas in Chipata two were under the age of five and one participant was over five, but below 15 years. The Kaplan Meier survival analysis of the PCR uncorrected data showed estimates of success of 1.00 between Day 0 to 6; 0.98 from day 7 to 20; 0.94 from day 21 to 27 and 0.9 on Day 28. The estimate of failure cumulative incidence was 0.00 from Day 0 to 6; 0.02 from Day 7 to 20; 0.06 from Day 21 to 27 and 0.1 on Day 28. The proportion of success and failure of the study participants at each point in time was not significant at the 95% (CI, 0.884, 1.026) and (−0.026, 0.116), respectively (Figure 2A).

**Table 3:** **Summary of classification of treatment outcomes (PCR uncorrected)**

Classification	Number	Proportion	Lower 95% CI	Upper 95% CI
**ETF**	0	0.000	0.000	0.021
**LCF**	6	0.035	0.013	0.075
**LPF**	12	0.070	0.037	0.119
**ACPR**	153	0.895	0.839	0.936
**Total analysis**	**171**			
**WTH**	0			
**LFU**	5	0.028		
**Total**	**176**			

*Abbreviations: ACPR* Adequate Clinical and Parasitological Response, *ETF* Early Treatment Failure, *LCF* Late Clinical Failure, *LPF* Late Parasitological Failure, *LFU* Loss to Follow Up, *WTH* Withdrawn.

PCR genotyped cure rate showed that 100% (CI 97.6 – 100, n = 153)) of the patients had ACPR to the AL treatment (Table 4). The Kaplan Meier survival analysis of the PCR corrected data showed estimates of success of 1.00 from day 0 to 28, translating into an estimate of failure cumulative incidence of 0.00 from day 0 to 28 (Figure 2B). The proportion of study participants with gametocytes on Day 0 was 0.5% (1/176) and by Day 7 was 1.1% (2/176). There were no gametocytes observed on Days 14 and 21. However on Day 28, 2 (1.1%) study participants had gametocytes. During the 28-day follow up no adverse events were observed.

**Table 4:** **Summary of Classification of treatment outcomes (PCR - corrected)**

Classification	Number	Proportion	Lower 95% CI	Upper 95% CI
**ETF**	0	0.000	0.000	0.024
**LCF**	0	0.000	0.000	0.024
**LPF**	0	0.000	0.000	0.024
**ACPR**	153	1.000	0.976	1.000
**Total analysis**	**153**			
**WTH**	18			
**LFU**	5	0.131		
**Total**	**176**			

*Abbreviations: ACPR* Adequate Clinical and Parasitological Response, *ETF* Early Treatment Failure, *LCF* Late Clinical Failure, *LPF* Late Parasitological Failure, *LFU* Loss to Follow Up, *WTH* Withdrawn.

### Secondary study outcomes

**Figure 3:**
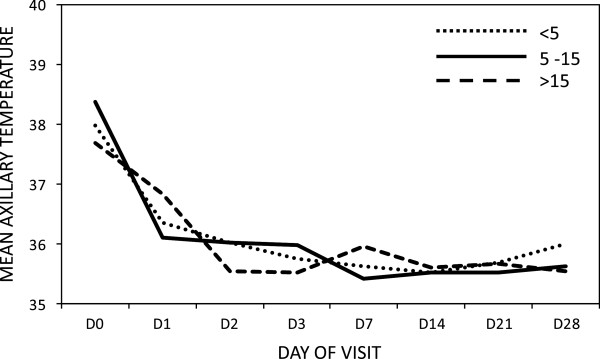
**Fever (axilliary temperature ≥37.5°C) clearance according to day of visit and age of study participant.**

**Figure 4:**
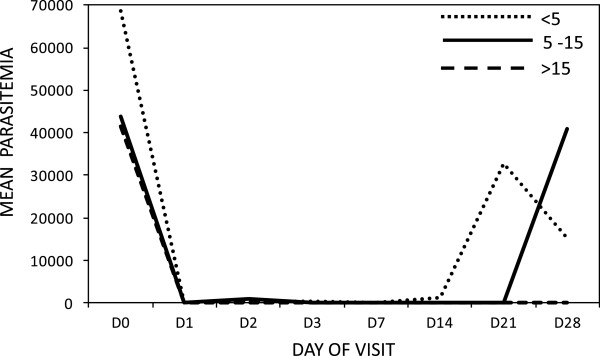
**Parasitaemia clearance according to day and age of study participant.**

A hundred percent (100%) reduction of mean parasitaemia was observed in all age groups on Day 1. However, on Day 2 under five children showed 99.7% and the 5 to 15 age groups showed 98% decline. *Plasmodium falciparum* parasitaemia were recorded in the under-five age group on days 3 to day 28 as follows: Day3: 2.2% (2/90), Day 7: 3.3% (3/90), Day 14: 1.1% (1/90) , Day 21: 6.7% (6/90) and Day 28: 4.4% (4/90). Likewise, parasitaemia was reported in the age group 5 to 15 on Day 3: 6.4% (4/63) and Day 28: 9.5% (6/63) (Figure 3). The two study participants who had parasitaemia on day 3 did not meet all the criteria required to be withdrawn from the study based on an ETF classification as the parasitaemia observed was less than that of day 0 and they were both afebrile. There was a general reduction in mean fever within 24 hours of initiation of treatment, which was maintained till the end of the 28-day follow up (Figure 4).

## Discussion

This study has demonstrated that fixed dose artemether-lumefantrine is effective for the treatment of uncomplicated *P. falciparum* malaria in all ages in areas with high malaria transmission intensity. The study comes nine years after its adoption as first-line anti-malarial in Zambia [5]. The high cure rates and tolerability of AL obtained in this study were consistent with those reported previously [12–14].

WHO recommends a review of treatment policy for uncomplicated malaria at 10% treatment failure [10]. This cut-off point was not reached in this study. Treatment failures have been associated primarily with either suboptimal plasma drug levels of the anti-malarial, re-infections or gene mutations resulting in parasite resistance [15, 16]. This study did not record any early treatment failure, suggesting that there may not be any need to include analysis of drug plasma levels in similar future studies [17]. However, the observation of asexual parasitaemia on days 3 and 7, in the predominately non-immune under-five age group raises concerns even though these infections were determined to be new infections. This finding requires further study to better understand transmission dynamics effects on parasitaemia persistence in a population post treatment. Slower parasite clearance *in vivo* to artemisinins has been reported elsewhere, particularly on the Thai-Cambodian border [18, 19].

Pre-treatment gametocytaemia was cleared by day 7, indicating that AL is still effective with regard to gametocyte clearance, and, suggesting a potential role in transmission reduction. Thus further suggesting a continued role in malaria control for therapeutic interventions, particularly with current calls for malaria elimination in most endemic countries [20, 21].

Zambia is among a few countries in the sub-region which has conducted or maintained regular studies in its sentinel sites in different areas across the country representing the different geographical and epidemiological profiles to monitor therapeutic efficacy of anti-malarial medicines. In the past three to four years efficacy studies faced a challenge on account of reduced malaria cases, which made it difficult and expensive to attain the required sample size. Therefore, to achieve the required sample size in this study, for the first time in Zambia, persons of all ages were enrolled rather than restricting the study to children under the age of five years as was the case previously [10]. Thus in part, the findings of this study reflect a detected efficacy influenced by previous exposure to malaria infection.

A key limitation of the study was the lack of a corresponding assessment in anaemia in the study participants, which would have provided data on longitudinal effects of malaria parasite infection in the study areas. An important strength of the study is that the study provides vital data collected in a high *P. falciparum* transmission (>15% parasitaemia) area in Zambia.

## Conclusion

Artemether-lumefantrine remains a safe and effective drug for the treatment of uncomplicated falciparum malaria in Zambia. AL is well tolerated in all ages when administered. The efficacy of this ACT needs to be carefully monitored periodically since treatment failures can occur due to resistance as well as sub- therapeutic levels due to non-compliance of therapeutic dosage. It is also important to strengthen routine monitoring of anti-malarial efficacy particularly in light of planned elimination efforts which will in part depend on therapeutic interventions.

## Abbreviations

AL: Artemether-lumefantrine
ACT: Artemisinin-based combination therapy
ACPR: Adequate clinical and parasitological response
ETF: Early treatment failures
LCF: Late clinical failure
LPF: Late parasitological failure
PCR: Polymerase chain reaction.
